# Tetra­aqua(2,2′-bipyridine-κ^2^ *N*,*N*′)magnesium(II) bis­(4-bromo­benzoate)

**DOI:** 10.1107/S1600536810041474

**Published:** 2010-10-23

**Authors:** Bi-Song Zhang, Chang-Sheng Wu, Wei Xu

**Affiliations:** a College of Material Science and Chemical Engineering, Jinhua College of Profession and Technology, Jinhua, Zhejiang 321017, People’s Republic of China; b Municipal Key Laboratory of Inorganic Materials Chemistry, Institute for Solid State Chemistry, Ninbo University, Ningbo, 315211, People’s Republic of China

## Abstract

In the complex cation of the title compound, [Mg(C_10_H_8_N_2_)(H_2_O)_4_](C_7_H_4_BrO_2_)_2_, the Mg^II^ atom is coordinated by two N atoms from a 2,2′-bipyridine ligand and four water O atoms in a distorted MgN_2_O_4_ octa­hedral geometry. The cation is located on a special position on a twofold rotation axis which passes through the Mg^II^ atom and the centroid of the 2,2′-bipyridine ligand. The 2,2′-bipyridine ligands exhibit nearly perfect coplanarity (r.m.s. deviation = 0.0035 Å) . In the crystal, O—H⋯O and C—H⋯O, C—H⋯Br hydrogen bonds and π–π stacking inter­actions [mean inter­planar distance of 3.475 (6) Å between adjacent 2,2′-bipyridine ligands] link the cations and anions into a three-dimensional supra­molecular network. One Br atom is disordered over two sites with occupancy factors of 0.55 and 0.45.

## Related literature

For related magnesium(II) complexes with 1.10-phenanthroline and pyridine ligands, see: Halut-Desportes (1981 ▶); Hao *et al.* (2008 ▶); Zhang (2004 ▶).
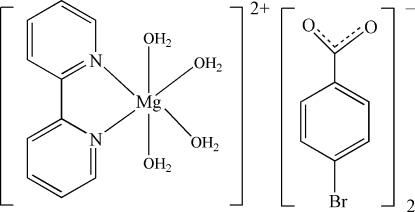


## Experimental

### 

#### Crystal data

[Mg(C_10_H_8_N_2_)(H_2_O)_4_](C_7_H_4_BrO_2_)_2_
*M* *_r_* = 652.56
Monoclinic, 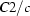

*a* = 30.275 (6) Å
*b* = 12.308 (3) Å
*c* = 7.5785 (15) Å
β = 103.90 (3)°
*V* = 2741.2 (11) Å^3^
*Z* = 4
Mo *K*α radiation
μ = 3.03 mm^−1^
*T* = 290 K
0.31 × 0.27 × 0.19 mm

#### Data collection

Rigaku R-AXIS RAPID diffractometer
Absorption correction: multi-scan (*ABSCOR*; Higashi, 1995 ▶) *T*_min_ = 0.406, *T*_max_ = 0.562
10517 measured reflections
2412 independent reflections
1505 reflections with *I* > 2σ(*I*)
*R*_int_ = 0.063

#### Refinement

*R*[*F* ^2^ > 2σ(*F* ^2^)] = 0.056
*wR*(*F* ^2^) = 0.174
*S* = 1.08
2412 reflections
171 parameters
1 restraint
H-atom parameters constrained
Δρ_max_ = 0.88 e Å^−3^
Δρ_min_ = −0.49 e Å^−3^

### 

Data collection: *RAPID-AUTO* (Rigaku, 1998 ▶); cell refinement: *RAPID-AUTO*; data reduction: *CrystalStructure* (Rigaku/MSC, 2002 ▶); program(s) used to solve structure: *SHELXS97* (Sheldrick, 2008 ▶); program(s) used to refine structure: *SHELXL97* (Sheldrick, 2008 ▶); molecular graphics: *SHELXTL* (Sheldrick, 2008 ▶); software used to prepare material for publication: *SHELXL97*.

## Supplementary Material

Crystal structure: contains datablocks I, New_Global_Publ_Block. DOI: 10.1107/S1600536810041474/rk2240sup1.cif

Structure factors: contains datablocks I. DOI: 10.1107/S1600536810041474/rk2240Isup2.hkl

Additional supplementary materials:  crystallographic information; 3D view; checkCIF report

## Figures and Tables

**Table 1:** Hydrogen-bond geometry (Å, °)

*D*—H⋯*A*	*D*—H	H⋯*A*	*D*⋯*A*	*D*—H⋯*A*
O1—H1*A*⋯O3^i^	0.82	1.89	2.702 (4)	172
O1—H1*B*⋯O3^ii^	0.82	1.84	2.640 (4)	165
O2—H2*A*⋯O4^iii^	0.82	1.86	2.679 (7)	175
O2—H2*B*⋯O3^ii^	0.82	2.08	2.790 (5)	145
C2—H2⋯Br1^iv^	0.93	3.00	3.525 (7)	117
C2—H2⋯Br1′^iv^	0.93	3.11	3.612 (7)	116
C3—H3⋯O4^v^	0.93	2.53	3.239 (6)	133

Symmetry codes: (i) 
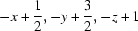
; (ii) 
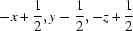
; (iii) 
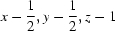
; (iv) 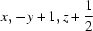; (v) 
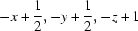
.

**Fig. 1.**
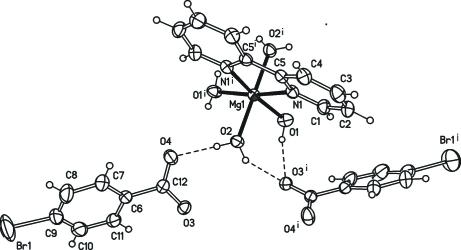
The molecule structure of the title compound showing the atom-labelling scheme. Displacement ellipsoids are drawn at the 40% probability level. Symetry code: (i) -x, y, -z+1/2. H atoms are presented as a small spheres of arbitrary radius. Only major position of Br1 atom is drawn. Selected hydrogen bonds are drawn by dashed lines.

**Fig. 2.**
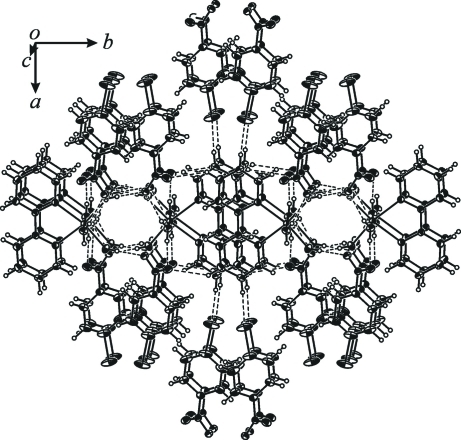
A packing diagram of the title complex, viewed down the c axis. The O—H···O, C—H···O and C—H···Br hydrogen bonds are drawn by dashed lines.

## supplementary crystallographic information

### Comment

Magnesium(II) ions with 1.10-phenanthroline (*phen*) and pyridine (*py*) ligands can form tetraaqua(*L*)_n_Magnesium(II) (*L* = *phen*, n = 1; *L* = *py*, n = 2) complex cation ( Halut-Desportes, 1981; Hao *et al.*, 2008; Zhang, 2004). In this paper we report synthesis and structure of the title compound. The crystal structure of title compound consists of [Mg(H_2_O)_4_(2,2'-*bipy*)]^2+^ complex cations and 4-bromobenzoate anion (Fig. 1). The cation placed in special position on twofold axis, which passes through Mg^II^ atom and middle C5—C5^i^ bond of 2,2'-*bipy* molecule. Symmetry code: (i) -*x*, *y*, -*z*+1/2. In the cation, the Mg^II^ atom is coordinated by two N atoms from one 2,2'-*bipy* ligands, four O atoms from four different water molecules, completing a distorted MgN_2_O_4_ octahedral geometry. The Mg—N bond length is 2.199 (4)Å and Mg—O bond lengths are 2.035 (3)Å and 2.042Å. The chelating *bipy* ligands exhibit nearly perfect coplanarity (r.m.s. deviations = 0.0035Å ). The mean interplanar distances of 3.475 (6)Å between adjacent *bipy* ligands indicate π···π stacking interactions (Fig. 2). The complex cations and 4-bromobenzoate anins are connected *via*π···π stacking interactions and O—H···O and C—H···O, C—H···Br hydrogen bonds (Table 1) into a three-dimensional supramolecular network.

### Experimental

MgCl_2_^.^6H_2_O (0.11 g, 0.54 mmol) was dissolved in appropriate amount of water, and then 1M Na_2_CO_3_ solution was added. MgCO_3_ was obtained by filtration, which was then washed with distilled water for 5 times. The freshly prepared MgCO_3_, 4-bromobenzoic acid (0.0508 g, 0.24 mmol), 2,2'-bipyridine (*bipy*) (0.0394 g, 0.22 mmol ), CH_3_OH/H_2_O (v/v = 1:2, 15 ml) were mixed and stirred for 2.0 h. Subsequently, the resulting cream suspension was heated in a 23 ml teflon-lined stainless steel autoclave at 433 K for 5800 minutes. After the autoclave was cooled to room temperature according to the procedure at 2600 minutes. the solid was filtered off. The resulting filtrate was allowed to stand at room temperature, and slow evaporation for 4 months afforded colourless block single crystals.

### Refinement

C-bound H atoms were placed in calculated positions, with C—H = 0.93Å and *U*_iso_(H) = 1.2*U*_eq_(C), and were refined using the riding-model approximation. The H atoms of the water molecule were located in a difference Fourier map and refined with an O—H distance restraint of 0.82 (1)Å and *U*_iso_(H) = 1.5*U*_eq_(O). The Br1 atom during anisotropic refinement procedure became prolate and was splitted on two positions with occupancy factors of 0.55 and 0.45.

### Crystal data

**Table:**

[Mg(C_10_H_8_N_2_)(H_2_O)_4_](C_7_H_4_BrO_2_)_2_	*F*(000) = 1312
*M**_r_* = 652.56	*D*_x_ = 1.581 Mg m^−^^3^
Monoclinic, *C*2/*c*	Mo *K*α radiation, λ = 0.71073 Å
Hall symbol: -C 2yc	Cell parameters from 6277 reflections
*a* = 30.275 (6) Å	θ = 3.2–25.0°
*b* = 12.308 (3) Å	µ = 3.03 mm^−^^1^
*c* = 7.5785 (15) Å	*T* = 290 K
β = 103.90 (3)°	Block, colourless
*V* = 2741.2 (11) Å^3^	0.31 × 0.27 × 0.19 mm
*Z* = 4	

### Data collection

**Table:**

Rigaku R-AXIS RAPID diffractometer	2412 independent reflections
Radiation source: fine-focus sealed tube	1505 reflections with *I* > 2σ(*I*)
graphite	*R*_int_ = 0.063
ω–scans	θ_max_ = 25.0°, θ_min_ = 3.2°
Absorption correction: multi-scan (*ABSCOR*; Higashi, 1995)	*h* = −35→35
*T*_min_ = 0.406, *T*_max_ = 0.562	*k* = −14→14
10517 measured reflections	*l* = −9→7

### Refinement

**Table:**

Refinement on *F*^2^	Primary atom site location: structure-invariant direct methods
Least-squares matrix: full	Secondary atom site location: difference Fourier map
*R*[*F*^2^ > 2σ(*F*^2^)] = 0.056	Hydrogen site location: inferred from neighbouring sites
*wR*(*F*^2^) = 0.174	H-atom parameters constrained
*S* = 1.08	*w* = 1/[σ^2^(*F*_o_^2^) + (0.0695*P*)^2^ + 6.5977*P*] where *P* = (*F*_o_^2^ + 2*F*_c_^2^)/3
2412 reflections	(Δ/σ)_max_ < 0.001
171 parameters	Δρ_max_ = 0.88 e Å^−^^3^
1 restraint	Δρ_min_ = −0.49 e Å^−^^3^

### Special details

**Table:**

Geometry. All s.u.'s (except the s.u. in the dihedral angle between two l.s. planes) are estimated using the full covariance matrix. The cell s.u.'s are taken into account individually in the estimation of s.u.'s in distances, angles and torsion angles; correlations between s.u.'s in cell parameters are only used when they are defined by crystal symmetry. An approximate (isotropic) treatment of cell s.u.'s is used for estimating s.u.'s involving l.s. planes.
Refinement. Refinement of *F*^2^ against ALL reflections. The weighted *R*-factor w*R* and goodness of fit *S* are based on *F*^2^, conventional *R*-factors *R* are based on *F*, with *F* set to zero for negative *F*^2^. The threshold expression of *F*^2^ > 2σ(*F*^2^) is used only for calculating *R*-factors(gt) etc. and is not relevant to the choice of reflections for refinement. *R*-factors based on *F*^2^ are statistically about twice as large as those based on *F*, and *R*-factors based on ALL data will be even larger.

### Fractional atomic coordinates and isotropic or equivalent isotropic displacement parameters (Å^2^)

**Table:**

	*x*	*y*	*z*	*U*_iso_*/*U*_eq_	Occ. (<1)
Mg1	0.0000	0.29391 (14)	0.2500	0.0366 (5)	
Br1	0.2137 (3)	0.9201 (5)	0.0828 (12)	0.1255 (19)	0.55
Br1'	0.2127 (4)	0.8733 (7)	0.0939 (16)	0.1255 (19)	0.45
N1	0.04459 (13)	0.1518 (3)	0.2599 (5)	0.0410 (9)	
O1	0.05190 (12)	0.4001 (2)	0.2564 (4)	0.0521 (9)	
H1A	0.0561	0.4561	0.3166	0.078*	
H1B	0.0512	0.4061	0.1480	0.078*	
O2	−0.01205 (11)	0.2973 (2)	−0.0269 (4)	0.0494 (8)	
H2A	−0.0376	0.3009	−0.0944	0.074*	
H2B	0.0036	0.3273	−0.0868	0.074*	
O3	0.43473 (11)	0.9050 (2)	0.5752 (4)	0.0480 (8)	
O4	0.40497 (12)	0.7957 (3)	0.7472 (5)	0.0646 (10)	
C1	0.08821 (17)	0.1562 (4)	0.2545 (6)	0.0514 (12)	
H1	0.1024	0.2237	0.2649	0.062*	
C2	0.11341 (18)	0.0657 (4)	0.2343 (7)	0.0543 (12)	
H2	0.1438	0.0717	0.2302	0.065*	
C3	0.09216 (19)	−0.0333 (4)	0.2203 (7)	0.0591 (13)	
H3	0.1081	−0.0959	0.2060	0.071*	
C4	0.04759 (18)	−0.0403 (4)	0.2274 (6)	0.0510 (12)	
H4	0.0332	−0.1074	0.2198	0.061*	
C5	0.02400 (15)	0.0542 (3)	0.2461 (5)	0.0393 (10)	
C6	0.35589 (17)	0.8637 (4)	0.4824 (6)	0.0466 (11)	
C7	0.32382 (19)	0.7824 (4)	0.4683 (7)	0.0623 (14)	
H7	0.3305	0.7209	0.5411	0.075*	
C8	0.2819 (2)	0.7917 (6)	0.3469 (9)	0.0837 (19)	
H8	0.2609	0.7353	0.3331	0.100*	
C9	0.2716 (2)	0.8849 (7)	0.2471 (8)	0.089 (2)	
C10	0.3022 (2)	0.9671 (7)	0.2613 (8)	0.089 (2)	
H10	0.2946	1.0301	0.1932	0.106*	
C11	0.34477 (19)	0.9563 (5)	0.3780 (7)	0.0640 (14)	
H11	0.3661	1.0117	0.3863	0.077*	
C12	0.40163 (16)	0.8535 (3)	0.6125 (6)	0.0423 (10)	

### Atomic displacement parameters (Å^2^)

**Table:**

	*U*^11^	*U*^22^	*U*^33^	*U*^12^	*U*^13^	*U*^23^
Mg1	0.0387 (12)	0.0345 (10)	0.0359 (10)	0.000	0.0074 (9)	0.000
Br1	0.0583 (7)	0.219 (6)	0.0842 (12)	0.038 (3)	−0.0128 (6)	0.011 (3)
Br1'	0.0583 (7)	0.219 (6)	0.0842 (12)	0.038 (3)	−0.0128 (6)	0.011 (3)
N1	0.043 (2)	0.0397 (19)	0.0380 (19)	0.0001 (17)	0.0041 (16)	0.0003 (15)
O1	0.064 (2)	0.0470 (17)	0.0468 (18)	−0.0141 (15)	0.0161 (17)	−0.0094 (14)
O2	0.047 (2)	0.0625 (19)	0.0367 (16)	−0.0069 (16)	0.0061 (14)	0.0051 (14)
O3	0.045 (2)	0.0515 (17)	0.0492 (18)	−0.0063 (15)	0.0136 (15)	−0.0062 (14)
O4	0.052 (2)	0.078 (2)	0.056 (2)	−0.0141 (19)	−0.0018 (17)	0.0225 (18)
C1	0.042 (3)	0.054 (3)	0.057 (3)	−0.004 (2)	0.008 (2)	0.000 (2)
C2	0.040 (3)	0.062 (3)	0.060 (3)	0.015 (2)	0.012 (2)	0.003 (2)
C3	0.060 (4)	0.054 (3)	0.059 (3)	0.016 (3)	0.007 (3)	−0.002 (2)
C4	0.053 (3)	0.042 (2)	0.055 (3)	0.004 (2)	0.008 (2)	−0.001 (2)
C5	0.042 (3)	0.041 (2)	0.033 (2)	0.0058 (19)	0.0037 (19)	0.0007 (18)
C6	0.041 (3)	0.059 (3)	0.040 (2)	0.006 (2)	0.011 (2)	0.002 (2)
C7	0.049 (3)	0.073 (3)	0.063 (3)	−0.010 (3)	0.011 (3)	−0.002 (3)
C8	0.050 (4)	0.122 (5)	0.076 (4)	−0.020 (4)	0.008 (3)	−0.023 (4)
C9	0.046 (4)	0.164 (7)	0.054 (3)	0.023 (4)	0.009 (3)	0.008 (4)
C10	0.063 (4)	0.140 (6)	0.067 (4)	0.035 (4)	0.023 (3)	0.039 (4)
C11	0.052 (3)	0.081 (4)	0.062 (3)	0.009 (3)	0.019 (3)	0.022 (3)
C12	0.040 (3)	0.042 (2)	0.046 (3)	−0.004 (2)	0.013 (2)	−0.009 (2)

### Geometric parameters (Å, °)

**Table:**

Mg1—O1	2.035 (3)	C2—C3	1.371 (7)
Mg1—O1^i^	2.035 (3)	C2—H2	0.9300
Mg1—O2	2.042 (3)	C3—C4	1.366 (7)
Mg1—O2^i^	2.042 (3)	C3—H3	0.9300
Mg1—N1^i^	2.199 (4)	C4—C5	1.390 (6)
Mg1—N1	2.199 (4)	C4—H4	0.9300
Mg1—H1B	2.3424	C5—C5^i^	1.468 (9)
Br1—C9	1.939 (10)	C6—C7	1.380 (7)
Br1'—C9	1.885 (11)	C6—C11	1.382 (7)
N1—C1	1.332 (6)	C6—C12	1.500 (7)
N1—C5	1.346 (5)	C7—C8	1.382 (8)
O1—H1A	0.8200	C7—H7	0.9300
O1—H1B	0.8200	C8—C9	1.367 (9)
O2—H2A	0.8199	C8—H8	0.9300
O2—H2B	0.8201	C9—C10	1.358 (10)
O3—C12	1.274 (5)	C10—C11	1.384 (8)
O4—C12	1.228 (5)	C10—H10	0.9300
C1—C2	1.379 (7)	C11—H11	0.9300
C1—H1	0.9300		
			
O1—Mg1—O1^i^	100.12 (19)	C1—C2—H2	121.1
O1—Mg1—O2	87.58 (13)	C4—C3—C2	120.2 (5)
O1^i^—Mg1—O2	90.94 (13)	C4—C3—H3	119.9
O1—Mg1—O2^i^	90.94 (13)	C2—C3—H3	119.9
O1^i^—Mg1—O2^i^	87.58 (13)	C3—C4—C5	119.2 (4)
O2—Mg1—O2^i^	177.69 (19)	C3—C4—H4	120.4
O1—Mg1—N1^i^	167.24 (14)	C5—C4—H4	120.4
O1^i^—Mg1—N1^i^	92.61 (13)	N1—C5—C4	121.0 (4)
O2—Mg1—N1^i^	91.39 (13)	N1—C5—C5^i^	116.2 (2)
O2^i^—Mg1—N1^i^	90.45 (13)	C4—C5—C5^i^	122.8 (3)
O1—Mg1—N1	92.61 (13)	C7—C6—C11	118.9 (5)
O1^i^—Mg1—N1	167.24 (14)	C7—C6—C12	120.8 (4)
O2—Mg1—N1	90.45 (13)	C11—C6—C12	120.3 (4)
O2^i^—Mg1—N1	91.39 (13)	C6—C7—C8	120.5 (5)
N1^i^—Mg1—N1	74.7 (2)	C6—C7—H7	119.7
O1—Mg1—H1B	20.1	C8—C7—H7	119.7
O1^i^—Mg1—H1B	100.5	C9—C8—C7	119.3 (6)
O2—Mg1—H1B	67.5	C9—C8—H8	120.4
O2^i^—Mg1—H1B	111.0	C7—C8—H8	120.4
N1^i^—Mg1—H1B	155.1	C10—C9—C8	121.4 (6)
N1—Mg1—H1B	91.7	C10—C9—Br1'	129.1 (6)
C1—N1—C5	118.5 (4)	C8—C9—Br1'	109.5 (6)
C1—N1—Mg1	124.9 (3)	C10—C9—Br1	112.2 (6)
C5—N1—Mg1	116.0 (3)	C8—C9—Br1	126.4 (7)
Mg1—O1—H1A	124.7	Br1'—C9—Br1	17.5 (4)
Mg1—O1—H1B	101.6	C9—C10—C11	119.4 (6)
H1A—O1—H1B	116.4	C9—C10—H10	120.3
Mg1—O2—H2A	123.4	C11—C10—H10	120.3
Mg1—O2—H2B	126.4	C6—C11—C10	120.4 (6)
H2A—O2—H2B	102.4	C6—C11—H11	119.8
N1—C1—C2	123.4 (5)	C10—C11—H11	119.8
N1—C1—H1	118.3	O4—C12—O3	124.1 (4)
C2—C1—H1	118.3	O4—C12—C6	118.3 (4)
C3—C2—C1	117.7 (5)	O3—C12—C6	117.6 (4)
C3—C2—H2	121.1		

Symmetry codes: (i) −*x*, *y*, −*z*+1/2.

### Hydrogen-bond geometry (Å, °)

**Table:**

*D*—H···*A*	*D*—H	H···*A*	*D*···*A*	*D*—H···*A*
O1—H1A···O3^ii^	0.82	1.89	2.702 (4)	172
O1—H1B···O3^iii^	0.82	1.84	2.640 (4)	165
O2—H2A···O4^iv^	0.82	1.86	2.679 (7)	175
O2—H2B···O3^iii^	0.82	2.08	2.790 (5)	145
C2—H2···Br1^v^	0.93	3.00	3.525 (7)	117
C2—H2···Br1'^v^	0.93	3.11	3.612 (7)	116
C3—H3···O4^vi^	0.93	2.53	3.239 (6)	133

Symmetry codes: (ii) −*x*+1/2, −*y*+3/2, −*z*+1; (iii) −*x*+1/2, *y*−1/2, −*z*+1/2; (iv) *x*−1/2, *y*−1/2, *z*−1; (v) *x*, −*y*+1, *z*+1/2; (vi) −*x*+1/2, −*y*+1/2, −*z*+1.
